# Cancer incidence near municipal solid waste incinerators in Great Britain. Part 2: histopathological and case-note review of primary liver cancer cases

**DOI:** 10.1054/bjoc.1999.1046

**Published:** 2000-02-01

**Authors:** P Elliott, N Eaton, G Shaddick, R Carter

**Affiliations:** Small Area Health Statistics Unit, Imperial College School of Medicine, St Mary's Campus, Norfolk Place, London, W2 1PG, UK; Royal Marsden Hospital, Sutton, Surrey, SM2 5PT, UK

**Keywords:** liver cancer, incinerators

## Abstract

We reported previously a 37% excess risk of liver cancer within 1 km of municipal incinerators. Of 119/235 (51%) cases reviewed, primary liver cancer was confirmed in 66 (55%) with 21 (18%) definite secondary cancers. The proportions of true primaries ranging between 55% and 82% (i.e. excluding secondary cancers) give revised estimates of between 0.53 and 0.78 excess cases per 10^5^per year within 1 km. © 2000 Cancer Research Campaign


British Journal Cancer 2000) **82**(5), 1103–1106
© 2000 Cancer Research Campaign
DOI: 10.1054/ bjoc.1999.1046, available online at http://www.idealibrary.com on IDEAL®

Short Communication

### Cancer incidence near municipal solid waste incinerators in Great Britain. Part 2: histopathological and case-note review of primary liver cancer cases

**P Elliott[1], N Eaton[1], G Shaddick[1] and R Carter[2]**

[1]Small Area Health Statistics Unit, Imperial College School of Medicine, St Mary's Campus, Norfolk Place, London W2 1PG, UK; [2]Royal Marsden Hospital, Sutton, Surrey SM2 5PT, UK

**Summary** We reported previously a 37% excess risk of liver cancer within 1 km of municipal incinerators. Of 119/235 (51%) cases reviewed, primary liver cancer was confirmed in 66 (55%) with 21 (18%) definite secondary cancers. The proportions of true primaries ranging between 55% and 82% (i.e. excluding secondary cancers) give revised estimates of between 0.53 and 0.78 excess cases per $10^5$ per year within 1 km. © 2000 Cancer Research Campaign

**Keywords**: liver cancer; incinerators

We reported previously a 37% excess of liver cancer cases within 1 km of 52 municipal solid waste incinerators in Great Britain, 1974–1986 (Elliott et al, 1996). This finding was based on routine cancer registry data, which may overestimate the true incidence of primary liver cancer because of mis-diagnosed secondary tumours (Doll and Peto, 1983). The aim of the present study was to validate, as far as possible, the diagnoses of primary liver cancer among cases included in the original report, in order to help determine the size of any true excess in the vicinity of municipal incinerators.

### MATERIALS AND METHODS

Diagnostic material and case notes were sought for 235 cases (155 males; 82 cases < 65 years) identified in our previous report. Three cases previously found not to have liver cancer were excluded, while another three subsequently located < 1 km of an incinerator were added. The 235 cases comprised all 87 at < 1 km, and random samples of 74 from each of 1–7.5 km and > 7.5 km.

Details of cases were obtained from the Office for National Statistics, the Information and Statistics Division of the Scottish Health Service and from the 12 regional cancer registries involved. For death certificate only cases, the nearest hospital to address at death was contacted.

Copies of histopathology reports and unstained slides from one representative tissue block of the original diagnostic material were requested from pathology departments. Slides were stained routinely with haematoxylin and eosin and with periodic acid Schiff-diastase (PAS-d). If tissue blocks were unavailable, the loan of existing stained slides (which were then anonymized) was sought.

*Received 24 March 1999*
*Revised 19 August 1999*
*Accepted 6 September 1999*

*Correspondence to:* P Elliott

Three histopathologists reviewed the slides independently. Where there was any disagreement, a case-conference was held, the slides were re-examined and a consensus view obtained. Details of the death certificates were provided, if needed, at the case-conferences as were case notes where available. The three reviewers remained blind to the location of cases throughout.

Material for diagnostic review was obtained for 94 cases (40%), of which 26 also had clinical notes available. For an additional 25 cases (11%) without histopathological material, copies of the medical records were obtained. This gave a total of 119/235 cases (51%) for review.

A hepatologist reviewed the medical records, blinded to the diagnosis from death certificates, histopathological review and location of the cases relative to incinerators. A clinical diagnosis of hepatocellular carcinoma was made based on at least two of: a confirmatory histopathological or cytological report; radiological evidence (including ultrasound); alpha-fetoprotein concentration in serum > 500 $\mu g\ l^{-1}$. Medical histories were also scrutinized for evidence of alcoholic liver disease and hepatitis B virus infection. Differences in proportions of cases with distance from incinerators were tested using $\chi^2$.

### RESULTS

Death certificate diagnoses of the 235 cases were as follows: 140 (60%) had a diagnosis of primary liver cancer (71 hepatocellular carcinoma/hepatoma, 21 cholangiocarcinoma, three sarcoma and 45 not otherwise specified (NOS)); for 33 (14%) liver cancer, NOS was recorded, while 46 (20%) recorded secondary liver cancers and 14 (6%) had other diagnoses. Two death certificates could not be obtained.

Table 1 shows the review diagnoses for the 119 cases. Among the 94 cases with histopathological review, 54 (57%) were classified as definite primary liver cancer; 21 (22%) were liver cancer, NOS, while 14 (15%) were secondary cancers. For five cases, no evidence of malignancy was seen in the reviewed material.

**Table 1** Diagnosis after review of histopathology and clinical notes from 119 cases

| Diagnosis after review (ICD code) | Histopathology review No. (%) | Case notes review No. (%) | Total (%) |
|---|---|---|---|
| Primary liver cancer | 54 (57) | 12 (48) | 66 (55) |
| Hepatocellular carcinoma (155.0) | 46[a] | 8 | 54[a] |
| Primary carcinoma (NOS) (155.0) | 1 | 1 | 2 |
| Angiosarcoma (155.0) | 2 | 1 | 3 |
| Leiomyosarcoma (155.0) | 1 | 0 | 1 |
| Cholangiocarcinoma (155.1) | 4 | 2 | 6 |
| Liver cancer (NOS)[b] | 21 (22) | 5 (20) | 26 (22) |
| Carcinoma | 6 | 3 | 9 |
| Adenocarcinoma | 15 | 2 | 17 |
| Secondary liver cancer | 14 (15) | 7 (28) | 21 (18) |
| Secondary carcinoma (NOS) | 1 | 5 | 6 |
| Secondary adenocarcinoma | 9 | 2 | 11 |
| Secondary neuroendocrine carcinoma | 3 | 0 | 3 |
| Secondary spindle cell sarcoma | 1 | 0 | 1 |
| No malignancy found | 5 (5) | 1 (4) | 6 (5) |
| Total | 94 | 25 | 119 |

NOS: Not otherwise specified. [a] One case was a mixed hepatocellular carcinoma and cholangiocarcinoma. [b] ICD: 197.8 (8th revision) and 155.2 (9th revision).

**Table 2** Death certificate diagnoses vs reviewed histopathological diagnoses (94 cases)

| Death certificate diagnoses | Reviewed diagnoses | | | | | | | | | | | |
|---|---|---|---|---|---|---|---|---|---|---|---|---|
| | Primary liver cancer (*n* = 54) | | | | | Liver cancer (NOS) (*n* = 21) | | Secondary liver cancer (*n* = 14) | | | | No malignancy (*n* = 5) |
| | HCC | CC | Carcinoma (NOS) | Angio-sarcoma | Leiomyo-sarcoma | Carcinoma (NOS) | AC | Carcinoma (NOS) | AC | Spindle cell sarcoma | NE | |
| Primary liver cancer | | | | | | | | | | | | |
| HCC, hepatoma or PLC (*n* = 54) | 38[a] | 1 | 1 | 2 | 1 | 2 | 3 | 0 | 3 | 0 | 1 | 2 |
| CC (*n* = 6) | 0 | 0 | 0 | 0 | 0 | 2 | 3 | 0 | 0 | 0 | 0 | 1 |
| Sarcoma (*n* = 2) | 0 | 0 | 0 | 0 | 0 | 1 | 0 | 0 | 0 | 1 | 0 | 0 |
| Liver cancer (NOS) (*n* = 14) | 3 | 2 | 0 | 0 | 0 | 1 | 6 | 0 | 2 | 0 | 0 | 0 |
| Secondary liver cancer | | | | | | | | | | | | |
| Carcinoma (NOS) (*n* = 10) | 2 | 0 | 0 | 0 | 0 | 0 | 2 | 1 | 2 | 0 | 2 | 1 |
| AC metastatic (*n* = 2) | 0 | 0 | 0 | 0 | 0 | 0 | 0 | 0 | 2 | 0 | 0 | 0 |
| No malignancy (*n* = 6) | 3 | 1 | 0 | 0 | 0 | 0 | 1 | 0 | 0 | 0 | 0 | 1 |
| Total (*n* = 94) | 46 | 4 | 1 | 2 | 1 | 6 | 15 | 1 | 9 | 1 | 3 | 5 |

PLC: primary liver cancer; HCC: hepatocellular carcinoma; CC: cholangiocarcinoma; Carcinoma (NOS): carcinoma, not otherwise specified; AC: adenocarcinoma; NE: neuroendocrine carcinoma. [a]Includes one case of mixed HCC/CC.

Table 2 compares the diagnoses following histopathological review with diagnoses recorded on death certificates. Of the 54 confirmed primary liver cancer cases, 43 were thus recorded on the corresponding death certificate (80% concordance). There was no mention of liver cancer on the death certificate for four cases. In all, death certificates recorded a total of 62 primary liver cancers, only five (8%) of which were considered to be definite secondary cancers on histopathological review.

For the 25 cases with clinical notes only, primary liver cancer was confirmed in 12 cases (48%), liver cancer, NOS in five (20%), and seven cases (28%) were diagnosed as secondary cancers (Table 1).

In total, 66 (55%, 95% confidence interval (CI) 47–64%) cases were confirmed as primary liver cancer, 26 (22%, 95% CI 14–29%) were classified as liver cancer, NOS, 21 (18%, 95% CI 11–24%) were metastatic tumours and there were six cases (5%, 95% CI 1–9%) with no evidence of malignancy (Table 1).

Table 3 shows the numbers and proportion of cases that were available for review and the reviewed diagnoses with distance from incinerators. There was a higher proportion of reviewed cases from 1–7.5 km (62%) than at < 1 km (43%) or from the rest of Great Britain (49%) ($P = 0.04$), but no evidence to suggest that the proportions of the reviewed diagnoses differed by distance ($P = 0.61$).

For the 54 cases of confirmed hepatocellular carcinoma, co-existing cirrhosis was identified in 25 on histopathological review and recorded in the clinical notes of a further eight cases. Associated factors could be identified in 13 of these 33 cases: alcohol (ten), hepatitis B virus infection (two) and primary biliary cirrhosis (one). There was no evidence, though the numbers were small, that either the proportion of hepatocellular carcinoma cases with cirrhosis, or the distribution of possible risk factors for cirrhosis and primary liver cancer, varied with distance from the incinerators (not shown).

 *© 2000 Cancer Research Campaign*

**Table 3** Summary of reviewed diagnoses, and proportions of cases available for review, by distance from municipal incinerators: numbers (per cent)

| **Reviewed diagnoses** | **Distance** | | | | | | | |
|---|---|---|---|---|---|---|---|---|
| | **< 1 km** | | **1–7.5 km** | | **Rest of GB** | | | |
| | **Histopathological review** | **Case notes review** | **Histopathological review** | **Case notes review** | **Histopathological review** | **Case notes review** | **Total** | **$\chi^2$ test** |
| Primary liver cancer | 19 (51%) | 5 (14%) | 20 (43%) | 4 (9%) | 15 (42%) | 3 (8%) | 66 (56%) | |
| Unspecified (primary or secondary) | 5 (14%) | 2 (5%) | 8 (17%) | 2 (4%) | 8 (22%) | 1 (3%) | 26 (22%) | |
| Not primary liver cancer[a] | 5 (14%) | 1 (3%) | 10 (22%) | 2 (4%) | 4 (11%) | 5 (14%) | 27 (23%) | *P*=0.61[b] |
| Total available for review/total number of cases (%) | 37/87 (43%) | | 46/74 (62%) | | 36/74 (49%) | | 119/235 (51%) | *P*=0.04 |

[a]Secondary liver cancer (14 cases) or no malignancy (five cases: two at < 1 km; two at 1–7.5 km; one from rest of GB) found in histopathological review, or on review of case notes (eight cases: seven secondary cancers, and one no malignancy from rest of GB). [b] Test of difference between proportions of primary liver cancer and other diagnoses by distance (histopathological and case review diagnoses combined).

The histopathological review identified two cases of angiosarcoma of the liver within 7.5 km (at < 1 km and 3.8 km), both initially diagnosed as hepatocellular carcinoma. In contrast with primary liver cancer cases held on the national register, both cases had undergone extensive scrutiny in our study. The two cases were located around different incinerators. Neither was found to be an industrial case.

### DISCUSSION

In view of the small numbers, the present study had low power to address questions of *relative* risk of primary liver cancer associated with residence near incinerators, although it could address *absolute* risk. This is because, in the absence of any obvious trends in patterns of diagnosis of primary liver cancer and associated risk factors (such as cirrhosis) with distance from incinerators, it has to be assumed that any deficiencies in the registration system will affect both numerator (cases) and denominator (expected numbers) equally, leaving estimates of relative risk unchanged. By contrast, any tendency for the numbers of primary liver cancer cases to be overestimated in the routine data (as we and others have found), would give high estimates of the absolute numbers of excess cases.

A range of possible estimates of the excess risk can be made. If our estimate of 55% primary liver cancer cases is correct, the excess number reported previously of 23 cases < 1 km over a 13-year period is reduced to 12.6, and it is 18.8 when only definite secondary cancer cases (18%) are excluded, i.e. 0.53 and 0.78 excess cases per $10^5$ per year respectively. We would expect the true number of excess cases to lie somewhere between the two.

One difficulty in interpreting these numbers is the issue of socio-economic confounding (Jolley et al, 1992; Carstairs, 1995). As illustrated in Figure 1, registered cases of primary liver cancer in Great Britain are strongly related to deprivation – the figure shows more than twofold variation in risk between the most affluent areas and the most deprived. Despite adjustment for deprivation in our previous analyses, the possibility of ‘residual’ confounding could not be excluded (Elliott et al, 1996).

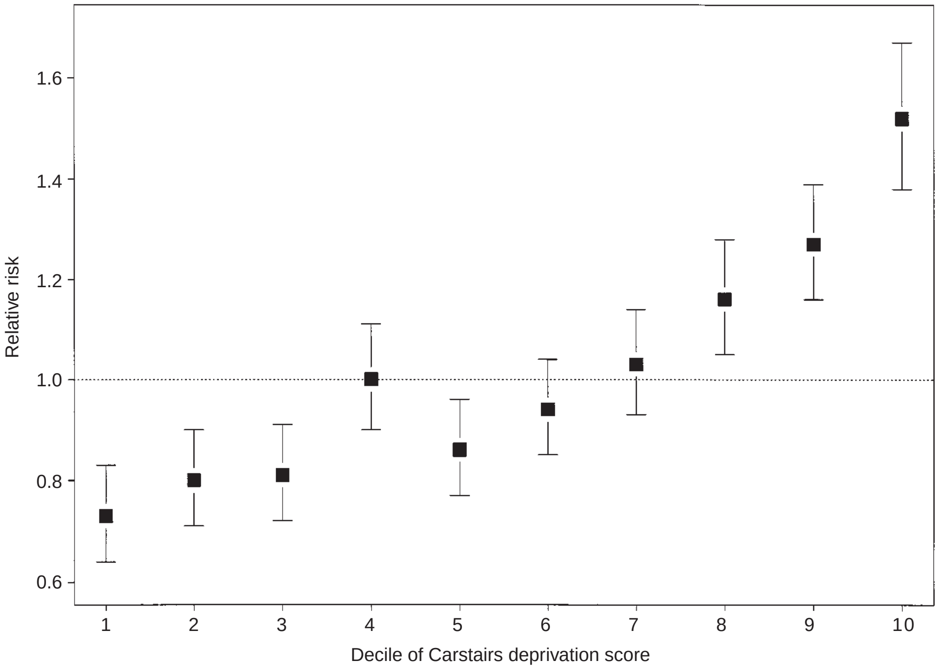

**Figure 1** Relative risk (95% CI) of primary liver cancer in Great Britain, 1980–1982, by quintile of scores on the Carstairs deprivation index at census ED level (1 = affluent, 10 = deprived)

*© 2000 Cancer Research Campaign*

Histopathological material or case notes could only be obtained for half of the 235 cases included here. Since some of them date back to 1974, material for many cases was either lost or discarded. Nonetheless, we have no reason to suppose that the sample of cases reviewed was materially different to the remainder, other than being diagnosed slightly later in the study period.

The 55% of registered primary liver cancer cases that were confirmed following diagnostic review is similar to a previous study in Great Britain that confirmed 62% of registered cases as primary liver cancer (Jenkins et al, 1995). Other investigations have found higher levels of agreement between cancer registration and histopathological diagnosis (Donato et al, 1995; Kaczynski, 1996). A relatively high proportion (80%) of confirmed primary liver cancers were recorded as such on the death certificate, while 69% (43/62) of death certificate diagnoses of primary liver cancer were confirmed on histopathological review. A large US study found that only 57% of confirmed primary liver cancers were recorded on death certificates, although 78% of death certificate diagnoses of primary liver cancer were confirmed by the histopathological findings (Percy et al, 1990). Lower levels of concordance have been reported in other studies (Cameron and McGoogan, 1981; Gobbato, 1982).

Evidence linking increased cancer incidence with emissions from incinerators is weak and indirect (Institute for Environment and Health, 1997). The findings in this and our previous paper (Elliott et al, 1996), if causal, relate to historical exposure patterns around incinerators. Since our original report, municipal solid waste incinerators in the UK have been required to meet emission limits in two European Communities' (1989*a*, *b*) directives and a dioxin emission limit of 1 ng $m^{-3}$ from December 1996. Consequently, there are now only 11 municipal solid waste incinerators currently in operation in the UK burning around 2.5 million tonnes of waste a year.

### ACKNOWLEDGEMENTS

We are grateful to Professor Peter Anthony (Exeter) and Professor Geraint Williams (Cardiff) for taking part (with Professor Carter) in the histopathological review panel and to Professor Howard Thomas (St Mary's Hospital, London) for reviewing the case notes. We thank the Office for National Statistics, the Information and Statistics Division of the Scottish Health Service, and the regional cancer registries for providing and checking details of cases included on the national cancer registries. We are grateful to Dr John Tomenson (ICI) and Mr Damien McElvenny (Health and Safety Executive) for checking details of angiosarcoma cases against their records of occupational cases. The Small Area Health Statistics Unit is funded by a grant from the Department of Health, Department of the Environment, Transport and The Regions, Health and Safety Executive, Scottish Office Home and Health Department, Welsh Office, and Northern Ireland Department of Health and Social Services. This work was also supported, in part, by an equipment grant from the Welcome Trust (0455051/Z/95/Z) The views expressed in this publication are those of the authors and not necessarily of the funding departments.

### REFERENCES

Cameron HM and McGoogan E (1981) A prospective study of 1152 hospital autopsies: I. Inaccuracies in death certificates. *Pathology* **133**: 273–283

Carstairs V (1995) The use and interpretation of deprivation indices in relation to health. *J Epidemiol Community Health* **49** (Suppl.2): S3–8

Doll R and Peto R (1983) Epidemiology of cancer. In: *Oxford Textbook of Medicine*, Weatherall DJ, Ledingham JGG and Warrell DA (eds), pp. 4.51–4.78. Oxford University Press: Oxford

Donato F, Rodella S, Chiesa R, Picocco C, Donati LF and Nardi G (1995) Diagnostic accuracy of primary liver cancer: implications for cancer registration. *Tumori* **81**: 86–90

Elliott P, Shaddick G, Kleinschmidt I, Jolley D, Walls P, Beresford J and Grundy C (1996) Cancer incidence near municipal solid waste incinerators in Great Britain. *Br J Cancer* **73**: 702–710

European Communities (1989*a*) Council Directive on the prevention of air pollution from new municipal waste incineration plants, 89/369/EEC. *Official Journal of the European Communities* **L163**: 32–36

European Communities (1989*b*) Council Directive on the prevention of air pollution from existing municipal waste incineration plants, 89/429/EEC. *Official Journal of the European Communities* **L203**: 50–54

Gobbato F, Vecchiet F, Barbierato D, Melato M and Manconi R (1982) Inaccuracy of death certificate diagnoses in malignancy: an analysis of 1405 autopsied cases. *Hum Pathol* **13**: 1036–1038

IARC (1997) Polychlorinated dibenzo-para-dioxins and polychlorinated dibenzofurans. In: *Monographs on the Evaluation of Carcinogenic Risk to Humans*, Vol 69. IARC, Lyon

Institute for Environment and Health (1997) *Health Effects of Waste Combustion Products*, Report R7. Institute of Environment and Health: Leicester

Jenkins D, Gilmore IT, Doel C and Allivans G (1995) Liver biopsy in the diagnosis of malignancy. *Q J Med* **88**: 819–825

Jolley DJ, Jarman B and Elliott P (1992) Socio-economic confounding. In: *Geographical and Environmental Epidemiology: Methods for Small-area Studies*, Elliott P, Cuzick J, English D and Stern R (eds), pp. 115–124. Oxford University Press: Oxford

Kaczynski J, Hansson G and Wallerstedt S (1996) Incidence of primary liver cancer and aetiological aspects: a study of a defined population from a low-endemicity area. *Br J Cancer* **73**: 128–132

Percy CL, Gloeckler RL and van Holten VD (1990) The accuracy of liver cancer as the underlying cause of death on death certificates. *Public Health Reps* **105**: 361–367

WHO (1967, 1977) *International Classification of Disease (ICD): Manual of the International Statistical Classification of Disease, Injuries, and Causes of Death*, 8th and 9th Revisions. WHO: Geneva

 *© 2000 Cancer Research Campaign*

